# Effects of a comprehensive structured patient education intervention on disease-related knowledge and behaviour change among people living with type 2 diabetes in the Philippines

**DOI:** 10.3389/fresc.2024.1374850

**Published:** 2024-02-28

**Authors:** Maria Rosan Trani, Imelda Bilocura, Shazna Bersabal, Rhea Karla Panilagao, Bj Rosos Toledo, Eduardo Garrido, Crystal Aultman, Paul Oh, Gabriela Lima de Melo Ghisi

**Affiliations:** ^1^ Cardiac Rehabilitation Unit, Chong Hua Hospital, Cebu City, Philippines; ^2^ Multidisciplinary Center Diabetes, Thyroid and Endocrine Center (DTEC), Chong Hua Hospital, Cebu City, Philippines; ^3^ Cardiovascular Prevention and Rehabilitation Program, University Health Network, Toronto, ON, Canada; ^4^ KITE Research Institute, University Health Network, Toronto, ON, Canada; ^5^ Department of Physical Therapy, Temerty Faculty of Medicine, University of Toronto, Toronto, ON, Canada

**Keywords:** diabetes mellitus type 2, patient education as topic, health knowledge, attitudes and practice, behavior change, Philippines

## Abstract

**Introduction:**

Addressing the ongoing needs of individuals with diabetes, particularly in low- and middle-income countries like the Philippines, requires a focus on regular follow-ups with healthcare teams, adherence to healthy behaviors, and effective patient education to prevent long-term complications. The aim of this study was to ascertain the impact of a comprehensive educational program for those living with diabetes in the Philippines.

**Methods:**

In a prospective study, a convenience sample of patients living with diabetes attending a cardiac rehabilitation or an outpatient diabetes clinic in the Philippines received a 12-week education intervention. Participants completed surveys at pre- and post-intervention assessing disease-related knowledge, health literacy, dietary habits, and tobacco use. Physical activity was measured by steps taken per day using wearable devices and by self-report of minutes of moderate or vigorous-intensity exercise per week. Satisfaction with the educational materials was also evaluated by a survey composed of Likert-type scale and open-ended questions. Descriptive statistics, paired *t*-tests or chi-square were used for data analysis.

**Results:**

Overall, 184 individuals living with diabetes type 2 (mean age = 54.4 ± 12.4, 32% female) completed both assessments. There was significant improvement in disease-related knowledge (*p* < 0.001), daily steps measured by a wearable device and self-reported minutes of moderate/vigorous-intensity exercise (*p* < 0.001), and the number of fruit and vegetable servings consumed per day (*p* = 0.001). No significant changes were observed in health literacy levels. One participant stopped using tobacco at post-education. Educational materials were highly satisfactory to participants. Lack of time, family responsibilities, and poor internet access were the main barriers to learning reported by participants. Suggestions to improve the education provided included assessment of information needs at the start of the education, having short summaries about the topics, follow-ups post-intervention, and inviting family members to sessions.

**Discussion:**

Results of this study demonstrated the positive effects a comprehensive structured patient education intervention on disease-related knowledge and behaviour changes among people living with type 2 diabetes in the Philippines.

## 1 Introduction

Type 2 diabetes mellitus poses a significant global health threat, with 1 in 11 adults worldwide affected by diabetes, according to the World Heart Federation (1, 2). The prevalence of diabetes is expected to rise substantially, reaching 629 million by 2045 from the current 425 million (2, 3). In the Philippines, the diabetes burden is substantial, with 7.1% of adults diagnosed in 2019, and it stands as the fourth leading cause of mortality in the country (4, 5). Despite the heightened risk of health complications, including cardiovascular disease, for individuals with diabetes, significant gaps persist in the care and prevention of this condition in the Philippines (6, 7).

One of the foremost challenges in contemporary diabetes care involves meeting the ongoing needs of individuals grappling with this chronic condition (8, 9). It is imperative to emphasize the significance of regular follow-ups with healthcare teams and the adherence to healthy behaviors to forestall potential long-term complications associated with diabetes (10). The process of adapting to life with a complex condition like diabetes is intricate and necessitates comprehensive patient education (11). While there is a growing body of evidence supporting the efficacy of patient education in managing chronic conditions, including diabetes (11–13), there is a notable dearth of information regarding effective programs tailored for individuals residing in low- and middle-income countries like the Philippines.

Cardiac rehabilitation (CR) plays a pivotal role in the comprehensive care and education of individuals with diabetes, particularly those at an elevated risk of cardiovascular complications (14). Beyond its traditional focus on heart health, CR programs have evolved to encompass a holistic approach that addresses the intricate interplay between diabetes and cardiovascular well-being (15). These programs provide a structured platform for educating diabetes patients about the synergistic relationship between diabetes management and cardiovascular health (14, 16). Through tailored educational interventions, individuals learn about the intricate nuances of diabetes, its impact on cardiovascular function, and strategies to mitigate associated risks. The emphasis is not only on disease-specific knowledge but also on fostering behavioral changes, such as adopting a heart-healthy lifestyle, adhering to medication and exercise regimens, and understanding the symbiotic connection between blood glucose control and cardiovascular outcomes (14). By integrating diabetes education into CR, healthcare providers can empower patients to take proactive steps in managing their overall health, thereby enhancing their quality of life and reducing the risk of complications (16).

A recent scoping review of diabetes education literature highlighted the scarcity of published research for individuals with diabetes in the Philippines, the available data indicated the effectiveness of patient education in enhancing diabetes knowledge, self-efficacy, anthropometric measures, A1C levels, utilization of healthcare services, and attitudes towards health (7). Regrettably, the review could not pinpoint a specific educational model suitable for this population, including in CR programs. Nonetheless, it underscored the imperative to conduct and disseminate further research on diabetes education in the Philippines.

In this context, we identified an evidence-based and comprehensive educational program—called Diabetes College—for those living with diabetes. Embedded within Health e-University, a virtual institute dedicated to promoting the prevention and management of chronic diseases, Diabetes College has undergone testing in high-income settings, demonstrating its efficacy in enhancing clinical outcomes, promoting heart-healthy behaviors, and augmenting knowledge in those individuals living with diabetes and participating in CR programs (17, 18). Therefore, the aim of this study was to ascertain the impact of this education intervention in those living with diabetes in the Philippines.

## 2 Methods

### 2.1 Design

This was a prospective observational study. Patients living with diabetes and attending a CR program or a diabetes outpatient clinic received the 12-week education intervention and completed pre- and post-intervention questionnaires. The study was conducted in the Chong Hua Hospital, Cebu City, Philippines following the standards required by the Declaration of Helsinki and approved by the Hospital's Ethics Committee (approval number: 2021-03). All participants provided written informed consent. Data were collected between November/2021 and August/2023.

### 2.2 Setting and intervention

Established in 2003, the CR unit at Chong Hua Hospital Heart Institute plays a vital role in the healthcare landscape, attending to an annual average of 250 patients, half of whom are managing diabetes. The phase 2 CR program spans 8 weeks, with patients participating in on-site sessions thrice weekly, each lasting for an hour. The dedicated healthcare team includes three cardiologists, one nurse, one physiotherapist, and one cardiology fellow. While the program primarily centers around exercise, it allocates 15 min per week for essential patient education. This educational component focuses on comprehending heart disease, procedures, medications, weight maintenance, risk factor modification, and dietary instructions. Notably, diabetes education was not integrated into the program before the commencement of this study.

The Diabetes Outpatient Program at the Diabetes, Thyroid, and Endocrine Center (DTEC) within Chong Hua Hospital was established in September 2021, offering care to 550 patients annually. Individuals managing diabetes participate in clinic visits once or twice a month, engaging in exercise and nutrition orientation sessions, as well as counseling. This comprehensive approach is facilitated by consultant endocrinologists on rotation, nurse educators, endocrine fellows, and dietitians, collectively contributing to the holistic management and support of patients with diabetes.

### 2.3 Educational intervention

The English version of Diabetes College was originally reviewed by 17 diabetes experts in the Philippines encompassing 3 physicians, 2 nurses, 2 pharmacists, 2 physical therapists, and 8 people who identified themselves as “hospital staff”. These experts offered valuable perspectives on how the materials could be structured for enhanced patient support. The consensus among the experts was that the materials were easily comprehensible and featured language and content culturally tailored to individuals residing in the Philippines. Additionally, the materials were reviewed by 4 patient partners, who were satisfied with the information provided and thought the resources were clear and useful.

In response to feedback provided by local experts and patients, materials from Diabetes College were structured into a 12-week educational curriculum (refer to Table 1). Study participants were provided with 12 weekly learning schedules containing hyperlinks to online resources, which included a comprehensive patient guide spanning 296 pages and a series of 12 animated videos (THRiVE series), each with an average duration of 5 min. The educational content was categorized into five knowledge pillars: managing diabetes, promoting physical activity, adopting healthy eating habits, enhancing overall well-being, and fostering self-management. The education process was designed to be self-directed, allowing patients the flexibility to engage with the learning materials according to their individual preferences and schedules. Rather than adhering to a rigid structure or timeline, patients had the autonomy to explore the educational content at a pace that suited their needs and learning styles. However, during the CR sessions or clinic visits, patients were encouraged to raise any questions they had regarding the content, and key messages were reiterated to reinforce understanding.

**Table 1:** 12-week educational schedule.

Session	Learning focus	Learning goals *By the end of this educational session, you will know:*	Resources
1	Create a plan for change	- How to choose one behaviour to focus on for the week - How to create an action plan for the week	- Diabetes college website - THRiVE video: create a plan for change - Diabetes patient guide: take control - Get active toolbox
2	Start an aerobic exercise program and exercise safety	- What aerobic exercise is and how to plan for exercise - The benefits of aerobic exercise - How to exercise safely - How your body may respond to a change in weather - How to prevent and treat hypoglycemia - How to prevent foot injuries	- Diabetes college website - THRiVE video: start an aerobic exercise program - Diabetes patient guide: getting active and starting an exercise program and your exercise safety - Tools and resources for getting active
3	Sit less, move more	- How sitting too much affects your health - Ways to sit less during the day	- Diabetes college website - THRiVE video: sit less, move more - Diabetes Patient guide: getting active and starting an exercise program
4	Start a resistance training program	- What resistance training is - The benefits of resistance training - How to do resistance training safely	- Diabetes College website - THRiVE video: start a resistance training program - Diabetes college patient guide: types of exercise - Get active toolbox
5	Choose healthy foods	- Types of foods that can help you improve your heart health and manage your blood sugar - How to use a nutrition facts table to choose healthy foods	- Diabetes college website - THRiVE video: choose healthy foods - Diabetes patient guide: nutrition basics
6	Develop a healthy relationship with food and eat the Mediterranean way	- The importance of paying attention to flavour, texture, and your surroundings when you eat - How to use the Hunger Scale to know when you are full - What foods to include in a heart healthy eating pattern	- Diabetes college website - THRiVE videos: develop a healthy relationship with food and eat the Mediterranean way - Diabetes patient guide: mindful eating and intuitive eating and cholesterol, triglycerides, and the Mediterranean diet pattern
7	Manage depression, stress, and burnout	- What depression, stress, and burnout are - Techniques you can try to help you feel in charge of your health	- Diabetes College website - THRiVE video: manage depression, stress, and burnout - Diabetes patient guide: sleep, stress, anxiety, and depression
8	Strengthen your social relationships	- How social relationships can improve your health - How heart disease or diabetes can affect sex and intimacy - Techniques to create healthy relationships	- Diabetes college website - THRiVE video: strengthen your social relationships - Diabetes patient guide: a healthy relationship
9	Take your medicine	- The common classes of heart medicines and how they help you - Common diabetes medicines and how they help you - Who can help you manage side effects and answer your questions	- Diabetes college website - THRiVE video: take your medicine - Diabetes patient guide: diabetes medicines
10	Health behaviours to prevent long-term problems with diabetes	- The health problems that can happen if your diabetes is not managed - Health behaviours that can prevent or delay these health problems	- Diabetes college website - Webinar: health behaviours to prevent long-term complications of diabetes - Diabetes patient guide: health problems with diabetes
11	Sleep well	- What might be stopping you from sleeping well - The signs of sleep apnea	- Diabetes college website - THRiVE video: sleep well - Diabetes patient guide: sleep, stress, anxiety, and depression
12	Choose health everyday	- How to maintain your healthy habits - What to do if you stop your healthy habit	- Diabetes college website - THRiVE video: choose health everyday - Diabetes patient guide: vision, goals, and action plans

### 2.4 Participants

A convenience sample of individuals diagnosed with type 2 diabetes was recruited for this study. Inclusion criteria comprised a confirmed diagnosis of diabetes type 2. Exclusion criteria encompassed individuals with limited proficiency in English, as well as those experiencing visual or cognitive impairments that might hinder survey completion. All participants were provided with internet cards to facilitate access to online resources.

### 2.5 Outcome measures

The following outcome measurements were assessed pre- and post-intervention: disease-related knowledge, health literacy, physical activity, dietary habits, and tobacco use. Participants also self-reported their work status, educational level, and marital status at pre-intervention. At post-intervention participants completed a satisfaction and usability survey about the use of educational materials, which included 10 Likert-type, yes/no and open-ended questions. Sociodemographic and clinical characteristics (age, gender, other diagnosis and comorbidities) were extracted from medical records.

Disease-related knowledge was assessed using the DiAbeTes Education Questionnaire (DATE-Q), which includes 20 true/false/I don't know items about self-management, long-term complications, being active, healthy eating, and psychosocial wellbeing (19). Higher scores indicate higher knowledge, with a maximum overall score of 20.

Health literacy was measured using the BRIEF Health Literacy Screening Tool, which asks respondents to complete four questions using a 5-point Likert-type scale (20). Scores range from 4 to 20, with the following health literacy skills categories: 4–12 (inadequate), 13–16 (marginal), and 17–20 (adequate).

Physical activity was assessed by self-report of minutes of moderate or vigorous-intensity exercise per week, and by daily step counts using a wearable device. The number of steps was recorded by asking participants to track steps for 7 consecutive days and record their numbers in a journal. Participants were categorized as active or inactive using a threshold of a mean of 10,000 steps per day over the seven-day period (21).

Dietary habits were assessed by self-reporting fruit and vegetable servings per day. Participants were categorized as following vs. not following the recommendations of consuming at least five servings of fruits and vegetables per day (22). Tobacco use was also self-reported (current, past, never).

### 2.6 Statistical analysis

SPSS Version 29.0 was used, and the level of significance was set at 0.05 for all tests. First, descriptive statistics were used to describe sociodemographic and clinical characteristics of participants (valid percentages are shown). Secondly, paired *t*-tests or chi-square tests were used to compare differences in characteristics. Third, changes in outcomes from pre- to post-interventions were assessed using paired *t*-tests or chi-square test by group (normality was met). Finally, results from the satisfaction/usability survey were reported using descriptive statistics and open-ended response items were coded using content analysis.

## 3 Results

### 3.1 Participant characteristics

A convenience sample of 184 individuals living with diabetes type 2 (mean age = 54.4 ± 12.4 years old, 32% female) accepted to be part of this study. Sociodemographic and clinical characteristics of the sample are shown in Table 2. Overall, participants were younger (i.e., less than 65 years old), male, married, employed, highly educated (i.e., college or university degree), and also had diagnosis of coronary artery disease. Participants attended a mean of 9.5 ± 1.0 educational sessions.

**Table 2:** Participants’ sociodemographic and clinical characteristics (*N* = 184).

Characteristics	*n* (%)
Sociodemographic	
Age	
Less than 65 years old	154 (83.7%)
65 years old or older	30 (16.3%)
Gender	
Male	125 (67.9%)
Female	59 (32.1%)
Marital status	
Married	154 (85.6%)
Single/Divorced	16 (8.9%)
Widowed	10 (5.5%)
Work status	
Employed	144 (78.7%)
Retired	22 (12.0%)
Unemployed	17 (9.3%)
Educational level	
High school or less	38 (21.1%)
College or University	142 (78.9%)
Clinical	
Coronary artery disease	134 (72.8%)
Hypertension	106 (57.6%)
Dyslipidemia	8 (4.3%)
Obesity	2 (1.1%)

Valid percentages report.

### 3.2 Effects of education

Mean scores of all outcome measures at each assessment are presented in Table 3. As shown, there was a significant increase from pre- to post-intervention in disease-related knowledge—total scores (*p* < 0.0001), as well as in all 5 knowledge areas. Being active was the area with the biggest increase in score pre- to post-education. Although significantly increased post-education, knowledge about self-management was the lowest among the 5 areas. No significant changes were observed in health literacy levels.

**Table 3:** Disease-related knowledge, health literacy, physical activity, dietary habits and tobacco use, pre- and post-education (*N* = 184).

Outcome measures	Score range	Pre-education	Post-education	Δ	*p*
Disease-related knowledge					
DATE-Q total scores (mean ± SD)	0–20	12.0 ± 2.9	15.4 ± 2.1	+3.4	<0.001
Subscale: self-management (mean ± SD)	0–4	1.2 ± 0.2	2.0 ± 0.9	+0.8	<0.001
Subscale: long-term complications (mean ± SD)	0–4	2.8 ± 1.0	3.5 ± 0.8	+0.7	<0.001
Subscale: being active (mean ± SD)	0–4	2.7 ± 0.9	3.7 ± 0.8	+1.0	<0.001
Subscale: healthy eating (mean ± SD)	0–4	2.9 ± 1.1	3.3 ± 0.7	+0.4	0.002
Subscale: psychosocial wellbeing (mean ± SD)	0–4	3.1 ± 1.0	3.6 ± 1.0	+0.5	<0.001
Health literacy					
BRIEF total scores (mean ± SD)	4–20	12.3 ± 2.2	12.5 ± 2.0	+0.2	0.84
Limited health literacy, *n* (%)	–	82 (44.6%)	84 (45.7%)	+2	–
Marginal health literacy, *n* (%)	–	101 (54.9%)	95 (51.6%)	−6	–
Adequate health literacy, *n* (%)	–	1 (0.5%)	5 (2.7%)	+4	–
Physical activity					
Steps taken per day (mean ± SD)	–	1,900.0 ± 1,122.9	6,075.0 ± 1,130.4	+4,175	<0.001
Active (i.e., reached ≥10,000 per day), *n* (%)	–	0 (0.0%)	0 (0.0%)	–	–
Minutes of moderate/vigorous-intensity exercise per week (mean ± SD)	–	98.7 ± 15.1	157.8 ± 91.5	+59.1	<0.001
Dietary habits					
Fruit and vegetable servings per day (mean ± SD)	–	2.3 ± 0.5	5.5 ± 1.7	+3.2	0.001
Consuming at least 5 fruits/vegetables per day, *n* (%)	–	12 (6.5%)	107 (58.2%)	+95	–
Tobacco use					
Current, *n* (%)		4 (2.7%)	3 (2.1%)	−1	–
Former, *n* (%)		3 (2.1%)	4 (2.7%)	+1	–
Never, *n* (%)		139 (95.2%)	139 (95.2%)	–	–

DATE-Q, diabetes education questionnaire; SD, standard deviation.

Valid percentages reported.

In regard to physical activity, daily steps measured by a device increased significantly at post-education (*p* < 0.001); however, none of the participants reached the recommended 10,000 steps per day. The self-reported minutes of moderate/vigorous-intensity exercise per week also significantly increased (*p* < 0.001). In regard to dietary habits, the number of fruit and vegetable servings per day increase significantly post-education (*p* = 0.001) and the percentage of participants who self-reported consuming at least 5 fruits or vegetables per day increased from 6.5% to 58.2%. Lastly, one participant stopped using tobacco.

### 3.3 Satisfaction and usability of the education

Table 4 presents the satisfaction and usability of educational resources (i.e., learning plans, THRiVE videos, and the patient guide). Overall, the majority of participants were satisfied with the resources provided, thought the resources were useful to help them manage diabetes, were often able to get information they were looking for, and were able to understand the information provided in each educational resource. Most participants also reported the amount of information in each education resource as “just right” and 52% indicated they accessed resources 1–4 times per month while attending the educational intervention.

**Table 4:** Satisfaction and usability of educational resources (*N* = 184).

	Overall	Learning plans	THRiVE videos	Patient guide
How satisfied are you with the educational resources? *n* (%)				
Very satisfied	–	53 (28.8%)	88 (47.8%)	50 (27.2%)
Satisfied	–	113 (61.4%)	77 (41.8%)	116 (63.0%)
Not satisfied	–	18 (9.8%)	18 (9.8%)	18 (9.8%)
I did not use it	–	0 (0.0%)	1 (0.5%)	0 (0.0%)
How useful were the educational resources to help you manage diabetes? *n* (%)				
Very useful	–	54 (29.3%)	89 (48.4%)	55 (29.9%)
Useful	–	130 (70.7%)	93 (50.5%)	129 (70.1%)
Not useful	–	0 (0.0%)	1 (0.5%)	0 (0.0%)
I did not use it	–	0 (0.0%)	1 (0.5%)	0 (0.0%)
How often were you able to get the information you were looking for in each educational resources? *n* (%)				
Always	–	20 (10.9%)	22 (12.0%)	56 (30.4%)
Sometimes	–	145 (78.8%)	143 (77.7%)	108 (58.7%)
Never	–	19 (10.3%)	18 (9.8%)	20 (10.8%)
I did not use it	–	0 (0.0%)	1 (0.5%)	0 (0.0%)
How often were you able to understand the information in each educational resources? *n* (%)				
Always	–	72 (39.1%)	95 (51.6%)	77 (41.8%)
Sometimes	–	112 (60.9%)	88 (47.8%)	107 (58.2%)
Never	–	0 (0.0%)	0 (0.0%)	0 (0.0%)
I did not use it	–	0 (0.0%)	1 (0.5%)	0 (0.0%)
How would you describe the amount of information in each educational resources? *n* (%)				
Too much	–	18 (9.8%)	20 (10.9%)	19 (10.3%)
Just right	–	166 (90.2%)	163 (88.6%)	162 (88.0%)
Too little	–	0 (0.0%)	0 (0.0%)	3 (1.6%)
I did not use it	–	0 (0.0%)	1 (0.5%)	0 (0.0%)
How many times did you use the educational resources per month? *n* (%)				
0 times	13 (7.1%)	–	–	–
1–4 times	95 (51.6%)	–	–	–
5–10 times	57 (31.0%)	–	–	–
More than 10 times	19 (10.3%)	–	–	–

In regard to content, participants indicated that all 5 knowledge pillars—treat diabetes, get active, eat healthy, feel well, and take control—were explained well, and highlighted the get active sessions as the ones with more impact on their health behaviours. When asked about what they would do if they needed information about a specific topic, 40% of participants reported they would look for answers in the educational materials provided.

Finally, answers from open-ended questions asking about information needs, barriers to learning, and suggestions for improvement were coded. Themes derived from the coded responses revealed persistent information gaps among participants concerning self-management and understanding the impact of diabetes on their quality of life and overall health. Participants identified time constraints, family responsibilities, and limited internet access as the primary barriers to effective learning. Notable suggestions to enhance the educational program included the importance of eliciting patients' specific needs prior to intervention delivery, facilitating the provision of concise summaries and key messages, implementing regular follow-ups with participants post-intervention, and incorporating family members into the educational sessions. These insights from participants offer valuable guidance for refining and optimizing future educational initiatives.

## 4 Discussion

Despite the increasing prevalence of diabetes in the Philippines, existing evidence highlights the suboptimal state of diabetes care and control, particularly in the realm of patient education. The outcomes of this first-ever study offering a comprehensive diabetes education program to individuals living with diabetes in the Philippines and attending CR, underscore the affirmative impact on disease-related knowledge, physical activity, and dietary habits. These findings are particularly heartening, as they emphasize the potential benefits of a culturally adapted, evidence-based, and patient-centered comprehensive education intervention. The study not only addresses a significant gap in diabetes education but also sheds light on the crucial necessity for Filipinos living with diabetes to acquire the knowledge and skills for effective self-management of their chronic condition. This pioneering effort paves the way for further initiatives aimed at enhancing diabetes care and education in the Philippines. The observed changes in disease-related knowledge and behaviors highlight the clinical significance of the educational intervention. Participants demonstrated significant increases in disease-related knowledge across all five knowledge areas, particularly in understanding the importance of physical activity. Improvements in physical activity levels, dietary habits, and tobacco cessation underscore the positive impact of the intervention on participants' ability to manage diabetes and promote better health outcomes.

It is essential to emphasize that the reasons behind the observed improvements in outcomes are likely influenced by multiple factors. The educational intervention, a key element of either CR or diabetes outpatient programs, involves providing counseling to participants. The CR program, specifically, stands out as a comprehensive secondary prevention initiative where patient education and counseling play integral roles (23). This program is designed to benefit patients dealing with multiple cardiovascular and chronic conditions, creating an avenue for valuable support (24). Within the CR program, individuals living with diabetes not only receive education but also gain access to an interprofessional healthcare team. This team structure allows for comprehensive care, and individuals with diabetes have the valuable opportunity to receive referrals to different specialists, thereby enhancing their self-management support (25). However, it is noteworthy that research has highlighted the importance of more than just the involvement of various healthcare team members in a patient's learning process. To ensure consistent and effective education for those living with type 2 diabetes, it is crucial to have the right resources and adopt a comprehensive approach (26). This approach ensures that individuals with diabetes receive education in a manner that is not only well-coordinated but also tailored to their specific needs and circumstances. The synergy of a well-structured program, an interprofessional team, and appropriate resources contributes to a more impactful and consistent educational experience for individuals managing type 2 diabetes.

The significance of health literacy in the realm of diabetes care cannot be overstated (27). Recently, the concept of health literacy has undergone a transformation, extending beyond the individual's skills in obtaining, processing, and understanding health information to encompass the demands placed on the health system in delivering information and instructions (28). In this context, this study did not observe an increase in health literacy post-education, which is in contrast to previous studies with people living with diabetes (29). The lack of change in health literacy post-intervention poses significant implications for the overall effectiveness of the educational program, potentially hindering participants' ability to understand and apply health information, sustain behavioral changes, and navigate healthcare systems effectively (29). This disparity might be attributed to inherent limitations in the measurement of health literacy, both within research methodologies and in the clinical setting. It underscores the need for a nuanced approach to comprehensively assess health literacy, considering not only individual capabilities but also the complex dynamics within the health system that influence the effective communication of information and instructions to individuals managing diabetes. Addressing these nuances is crucial for the development of targeted interventions that truly enhance health literacy in diabetes care. It is important to mention that while the incorporation of a health literacy assessment adds value to the study, the selection of the BRIEF Health Literacy Screening Tool warrants discussion regarding its suitability and inherent limitations (20). This tool is often chosen for its brevity and ease of administration, making it practical for use in clinical and research settings. However, it's important to acknowledge that the BRIEF tool provides only a brief assessment of health literacy and may not capture the full spectrum of health literacy skills. Its simplicity might overlook nuanced aspects of health literacy, such as the ability to comprehend complex health information or navigate healthcare systems effectively. Furthermore, the BRIEF Health Literacy Screening Tool may not be sensitive enough to detect variations in health literacy levels among different populations or cultural groups. Its generic nature may limit its applicability in diverse contexts where health literacy challenges may manifest differently.

There is a scarcity of information concerning patients' experiences, perspectives, and preferences in the realm of diabetes education (7, 29). The effectiveness of educational interventions is intricately influenced by the diversity of these experiences, views, and preferences, which can vary significantly both within and across individuals (30). Recognizing this variability is paramount, and cultural adaptation of materials is essential, alongside the provision of a range of resources to cater to these differences (31, 32). This study sought to address this gap by offering a diverse set of educational resources to individuals in the Philippines, meticulously reviewed by both experts and patients. The outcomes of this study not only shed light on patients' preferences regarding diabetes education but also extend an invitation to the broader research community to delve deeper into understanding the educational needs of individuals managing diabetes. The insights gleaned from this study underscore the importance of a patient-centered approach in diabetes education, encouraging ongoing exploration and refinement of educational strategies to better align with the diverse preferences and needs of the individuals involved.

Although our study provides favourable effects of a structured patient education on disease-related knowledge and behaviour changed among people living with type 2 diabetes and attending CR or diabetes programs in the Philippines, results should be interpreted with caution. Firstly, the absence of a comparison group (i.e., a group without education) precludes the drawing of causal conclusions. Moreover, the sustainability of the observed gains in knowledge and the adoption of healthy behaviors over time remains uncertain due to the lack of follow-up assessments. Secondly, the generalizability of results is limited to patients accessing CR and diabetes clinics, representing a minority of patients in the Philippines. Notably, the study sample was predominantly highly educated (79% with a college or university degree). This demographic composition poses a limitation to the generalizability of the study findings to a broader population. High levels of education within the sample suggest a potential bias towards individuals with greater access to resources, higher socioeconomic status, and potentially different health behaviors or attitudes compared to the general population. Consequently, the findings may not accurately reflect the experiences or perspectives of individuals with lower levels of education or socioeconomic status. Thirdly, the absence of structured clinical interviews to assess physical activity and dietary habits, relying solely on self-reported measures, introduces a potential limitation to the study results. Furthermore, it's important to note that this study utilized a convenience sample, and as such, power analysis was not conducted. The decision to employ a convenience sample implies that participants were selected based on availability and accessibility rather than through randomization or rigorous sampling methods. However, it's essential to acknowledge that convenience sampling can introduce selection bias, as individuals who are easily accessible may not be representative of the broader population under study. In addition, the incorporation of both objective measures (such as wearable devices for physical activity) and self-reported measures (via surveys) is advantageous; however, the self-reported data, especially concerning physical activity and dietary habits, has inherent limitations. Finally, critical outcome measures for individuals with diabetes, such as medication adherence and glycemic control, were not evaluated and should be included in future studies. Replication of the study in diverse patient samples across different regions of the Philippines, receiving varied care, is imperative for a more comprehensive understanding of the educational impact in this context.

In summary, the study revealed that implementing a comprehensive diabetes education intervention for individuals with type 2 diabetes in the Philippines led to significant improvements in disease-related knowledge and healthy behaviors, such as physical activity and dietary habits; while acknowledging limitations in generalizability, these promising findings underscore the importance of expanding educational resources and accessibility for Filipinos with diabetes. While acknowledging the study's limitations in terms of generalizability to a broader population, these promising findings underscore the critical importance of expanding educational resources and accessibility tailored to the unique needs of Filipinos living with diabetes. This study serves as a beacon, emphasizing the imperative to cultivate and disseminate effective educational initiatives to empower individuals with diabetes and enhance their overall well-being. Further research and strategic interventions are warranted to ensure the continued success and widespread impact of diabetes education in the Filipino context.

## Data Availability

The datasets presented in this article are not readily available because of restrictions imposed by the privacy of participants. Requests to access the datasets should be directed to Gabriela Ghisi gabriela.meloghisi@uhn.ca.
